# Medication adherence and blood pressure control amongst adults with primary hypertension attending a tertiary hospital primary care clinic in Eastern Nigeria

**DOI:** 10.4102/phcfm.v5i1.446

**Published:** 2013-02-25

**Authors:** Gabriel UP. Iloh, John N. Ofoedu, Patrick U. Njoku, Agwu N. Amadi, Ezinne U. Godswill-Uko

**Affiliations:** 1 Department of Family Medicine, Federal Medical Centre, Umuahia, Abia State, Nigeria; 2 Department of Public Health Technology, Federal University of Technology, Nigeria; 3 Department of Anaesthesiology, Federal Medical Centre, Umuahia, Abia State, Nigeria

## Abstract

**Background:**

As the case detection rates of hypertension increase in adult Nigerians, achieving target blood pressure (BP) control has become an important management challenge.

**Objectives:**

To describe medication adherence and BP control amongst adult Nigerians with primary hypertension attending a primary care clinic of a tertiary hospital in a resource-poor environment in Eastern Nigeria.

**Methods:**

A cross-sectional study was carried out in 140 adult patients with primary hypertension who have been on treatment for at least 6 months at the primary care clinic of Federal Medical Centre, Umuahia. A patient was said to have achieved goal BP control if the BP was < 140 per 90 mmHg. Adherence was assessed in the previous 30 days using a pretested researcher-administered questionnaire on 30 days of self-reported therapy. Adherence was graded using an ordinal scoring system of 0–4; an adherent patient was one who scored 4 points in the previous 30 days. Reasons for non-adherence were documented.

**Results:**

Adherence to medication and BP control rates were 42.9% and 35.0% respectively. BP control was significantly associated with medication adherence (*p* = 0.03), antihypertensive medication duration ≥3 years (*p* = 0.042), and taking ≥ one form of antihypertensive medication (*p* = 0.04). BP at the recruitment visit was significantly higher than at the end of the study (*p* = 0.036). The most common reason for non-adherence was forgetfulness (*p* = 0.046).

**Conclusions:**

The rate of BP control amongst the study population was low, which may be connected with low medication adherence. This study urges consideration of factors relating to adherence alongside other factors driving goal BP control.

## Abstrait

**Observance du traitement et contrôle de la pression artérielle chez des adultes souffrant d'hypertension primaire soignés dans un centre de soins primaires d'un hôpital tertiaire à l'Est du Nigéria**

**Contexte:**

Comme les taux de détection de cas d'hypertension augmentent chez les Nigérians d’âge adulte, le contrôle de la pression artérielle (Pa) est devenu un important défi à gérer.

**Objectifs:**

Décrire l'observance du traitement et le contrôle de la Pa chez les Nigérians d’âge adulte souffrant d'hypertension primaire traités dans un centre de soins primaires d'un hôpital tertiaire dans un environnement caractérisé par la rareté des ressources à l'Est du Nigéria.

**Méthodes:**

Une étude transversale a été réalisée auprès de 140 patients adultes souffrant d'hypertension primaire traitées depuis au moins 6 mois au centre de soins primaires du Centre médical fédéral d'Umuahia. Il est considéré qu'un patient a atteint son objectif de contrôle de la Pa si celle--ci est < 140 par 90 mmHg. L'observance a été évaluée dans les 30 jours précédents en utilisant un questionnaire prétesté administré par les chercheurs sur 30 jours d'autothérapie. L'observance a été notée en utilisant un système de notation ordinal allant de 0 à 4; un patient suivant à son traitement se voyait accorder une note de 4 dans les 30 jours précédents. Les raisons d'une mauvaise observance ont été documentées.

**Résultats:**

Les taux d'observance du traitement et de contrôle de la Pa s’élevaient respectivement à 42,9% et 35.0%. Le contrôle de la Pa est fortement associé à l'observance du traitement (*p* = 0.03), la durée du traitement antihypertensif ≥ 3 ans (*p* = 0.042), et la prise ≥d'une forme de traitement anti hypertensif (*p* = 0.04). Lors de la visite de recrutement, la Pa était nettement plus élevée qu’à la fin de l’étude (*p* = 0.036). La raison la plus courante de la non observance du traitement est l'oubli (*p* = 0.046).

**Conclusions:**

Le taux de contrôle de la Pa parmi la population étudiée était faible, ce qui peut avoir un rapport avec la faible observance du traitement. Cette étude pousse à prendre en considération les facteurs liés à l'observance ainsi qu'aux autres facteurs liés au contrôle de la Pa.

## Introduction

Hypertension is the most common cardiovascular disorder, posing a major clinical and public health challenge to populations in socio-economic and demographic transition.^1^ Studies have demonstrated that blood pressure (BP) tends to increase with age, and the relationship between BP and the risk of cardiovascular events is continuous, consistent and independent of other risk factors.^2, 3^ As the Nigerian population ages, the prevalence of hypertension is expected to increase unless effective preventive measures are implemented. Hypertension is therefore one of the most common causes of disability and death amongst the adult Nigerian population, and plays an important role in the causation of hypertensive heart failure, heart attack, arteriosclerosis, renal failure and stroke.^4^ Adequate control of BP was reported to reduce the incidence of stroke by an average of 35% – 40%, myocardial infarction by 20% – 25% and heart failure by more than 50%.^5^ For individuals of 40–70 years of age each increment of 20 mmHg in systolic BP or 10 mmHg in diastolic BP doubles the risk of cardiovascular disease across the entire BP range from 115 per 75 mmHg to 185 per 115 mmHg.^6^

Achieved BP is not synonymous with target or goal BP control, and the benefits of goal BP control have been elucidated in previous studies.^7, 8^ The primary focus of antihypertensive therapy is to achieve a goal BP of < 140 per 90 mmHg for the general population and < 130 per 80 mmHg for special high-risk populations such as patients with diabetes mellitus, renal disease and adverse cardiovascular events such as myocardial infarction and stroke.^2, 3^ The magnitude of BP control differs between the developing^9, 10, 11^ and developed countries.^12^ In Nigeria, BP control rates in adult patients range from 24.2% to 35.8% according to published studies.^9, 10, 11, 13, 14^ These disparities are probably due to a milieu of limited healthcare facilities, widespread personal and family poverty, ignorance, poverty of knowledge and other diverse factors.^15, 16^

Blood pressure control is influenced by multiple factors,^2, 3, 13, 15, 16, 17, 18^ some of which are related to the patients, the health professionals and government. Amongst the patient-related factors is adherence to antihypertensive medication. Adherence is one of the challenges of antihypertensive medication and is defined conceptually as the degree to which the patient conforms with treatment as prescribed.^19^ Several methods of measuring adherence to medication have been described.^20, 21, 22^ There is no ‘gold standard’ for precise measurement of adherence.^20^ Clinical measures include the use of questionnaires for 30 days of self-reported (self-administered) therapy. Pharmacological measures involve determination of serum and urinary concentrations of drugs or using biological markers integrated into the tablets.^21^ Although having higher sensitivity and specificity, pharmacological methods are not routinely used in clinical practice.^21^ Recently the medication event monitoring system (MEMS) was introduced.^22^ Assessment of adherence using the MEMS device involves microprocessor-based monitoring. Unfortunately the MEMS device is not readily available in the study area.

In order to provide necessary health services for management of hypertension in health facilities in Nigeria, clinicians should identify and determine the prevailing level of BP control and factors responsible for poor BP control. This study therefore provides additional evidence on the contribution of adherence factors to the growing problem of goal BP control in Nigeria. It is against this background that the researchers were motivated to study medication adherence and BP control amongst adults with primary hypertension attending a primary care clinic of a tertiary medical centre in a resource-poor environment in Eastern Nigeria.

## Ethical considerations

The Ethics Committee of the Federal Medical Centre (FMC), Umuahia, Abia State, Eastern Nigeria, granted approval for the study. Informed written consent was obtained from the respondents before recruitment into the study.

## Method

### Design

This was a clinic-based descriptive cross-sectional study carried out on 140 patients from April 2011 to November 2011 at the Department of Family Medicine of the FMC, Umuahia, which is a tertiary hospital in Abia State, Eastern Nigeria.

### Setting

Umuahia is the capital of Abia State in south-east Nigeria. The State is endowed with abundant mineral and agricultural resources, with a supply of professional, skilled, semi-skilled and unskilled manpower. Economic and social activities here are low compared to industrial and commercial cities such as Onitsha, Port Harcourt and Lagos. Until recently the capital city of Lagos and its environs have witnessed an upsurge in the number of banks, hotels, schools, markets, industries and fast food restaurants in addition to changing dietary and social lifestyles.

The FMC is located in the metropolitan city of Umuahia. This is a tertiary hospital established with the tripartite mandate of service delivery, training and research, and serves as a referral centre for primary and secondary public health institutions as well as missionary and private hospitals in Abia and the neighbouring states of Imo, Ebonyi, Rivers and Akwa Ibom. The Department of Family Medicine serves as a primary care clinic within the tertiary hospital setting of the medical centre. All adult patients, excluding those who need emergency healthcare services, paediatric patients and antenatal women, are first seen at the Department of Family Medicine, where diagnoses are made. Patients who need primary care are managed and followed up in the clinic, whilst those who need specialist care are referred to the respective core specialist clinics for further management. The clinic is run by consultant family physicians and postgraduate resident doctors in Family Medicine.

### Inclusion and exclusion criteria

The inclusion criteria were adult hypertensive patients aged ≥ 18 years who gave informed verbal consent, had been on outpatient treatment for hypertension in the clinic for at least 6 months, and who recorded at least three clinic visits (recruitment visit, penultimate visit before the end of study, and end of study visit). Exclusion criteria included critically ill patients, those with an established cause of hypertension, patients whose recommended^2, 3^ goal BP is < 130 per 80 mmHg, and special high-risk populations such as hypertensive patients with diabetes mellitus, renal disease and previous adverse cardiovascular events such as myocardial infarction and stroke. The eligible patients were consecutively recruited for the study.

### Sampling

Sample size estimation was determined using the formula^23^ for estimating minimum sample size for descriptive studies when studying proportions with an entire population size < 10 000 using an estimated population size of 200 adult hypertensive patients, based on the previous annual hypertensive patients’ attendance records at the clinic. These 200 adult hypertensives excluded other hypertensive patients referred to and being followed up in other medical outpatients’ clinics in the medical centre. The diabetic hypertensives were also excluded from this recorded number. The authors assumed that 50% of the adult hypertensives would have a good BP control rate and adhere to treatment, at a 95% confidence level with a 5% margin of error. This gave a sample estimate of 132 patients. A sample size of 140 adult hypertensive patients was selected to allow for attrition.

### Data collection methods

Adherence was assessed by use of a pretested, interviewer-administered questionnaire on 30 days of self-administered and reported therapy. Patients were seen at the recruitment visit, penultimate visit before the end of the study and then at the end of the study visit. At the end of study visit the adherence section of the data collection tool was administered. Information collected at the end of study visit included, (1) how many times per day do you take your BP medication?, (2) how many tablets do you take specific to your hypertensive condition?, (3) how often do you take your BP medication (all times, most times, sometimes, rarely, never), (4) how many dose(s) of your antihypertensive drugs have you missed in the previous one month? and (5) how much of your previous BP medication is remaining after the previous one-month visit? Adherence was graded using an ordinal scoring system of 0–4 points developed by the authors from a review of the literature^9, 10, 11, 12, 13, 14^ as follows: all the time = 4 points, most times = 3 points, sometimes = 2 points, rarely = 1 point, never = 0. Four points indicated adherence, whilst 0–3 points meant non-adherence. Reasons for non-adherence were documented for those who scored 0–3 points.

Pretesting of the adherence section of the data collection tool was done internally at the Family Medicine clinic of the FMC and externally at the Family Medicine clinic of the FMC in Owerri, Eastern Nigeria. Five diabetic hypertensive patients of the FMC in Umuahia were randomly used for pre-testing of the questionnaire, which lasted for one day, and five hypertensive patients were used in the FMC in Owerri. The pretesting was done to assess the applicability of the questionnaire tool internally and externally. All of the patients used for pretesting of the questionnaire instrument gave valid and reliable responses, confirming the clarity and applicability of the questionnaire tool, and questions were interpreted with the same meaning as intended. The questionnaire was administered by three resident doctors who were recruited and trained for the study, once to each eligible respondent at the end of study visit.

BP readings were based on the seventh report of the Joint National Committee on Prevention, Detection, Evaluation, and Treatment of High Blood Pressure classification and guidelines.^2^ The baseline BP was recorded at the time of recruitment (recruitment visit), subsequent BP was measured at the penultimate visit before the end of study visit, and then at the end of study visit. The duration of hypertension, class and number of antihypertensive drugs and reasons for non-adherence to antihypertensive medication were documented. Fasting blood sugar, urinalysis, urea and creatinine were done. Diagnosis of diabetes mellitus was based on a venous plasma glucose level of ≥ 126 mg/dL after an overnight fast, which was confirmed by a repeat test at the second clinic visit.^24^ The basic demographic variables of age, sex, marital status, education and occupation were also documented.

### Operational definitions

The authors defined hypertension as a systolic and/or diastolic BP ≥ 140 per 90 mmHg or documented use of antihypertensive medications in a person previously diagnosed with hypertension.^2^ A patient was defined as having goal BP control if his or her BP at the end of study visit was < 140 per 90 mmHg.^2, 3^ An adherent patient was defined as one who had a score of 4 points (took the prescribed doses of antihypertensive all the time) for the previous 30 days by the end of the study visit.

### Data analysis

The results generated were analysed using Statistical Package for Social Sciences (SPSS) software version 13.0 (Microsoft, Chicago, IL, United States of America [USA]) for calculation of percentages for categorical variables and means for continuous data. Means with standard deviations of continuous variables were generated where appropriate. Percentages and frequencies were compared by Chi-square analysis and the Student's *t*-test served to compare means. The level of statistical significance was set at *p* < 0.05.

## Results

The age of the hypertensive patients ranged from 32 to 83, years with mean age of 52 ± 7.4 years. There were 56 (40.0%) males and 84 (60.0%) females, with a male to female ratio of 1: 1.5 (Table 1).

**TABLE 1:** Basic demographic variables in the study population.

**Age (years)**	***n***	**%**
18–39	10	17.2
40–60	86	61.4
> 60	44	31.4
**Total**	**140**	**100.0**
**Sex**		
Male	56	40.0
Female	84	60.0
**Total**	**140**	**100.0**
**Marital status**		
Single	4	2.9
Married	101	72.1
Separated or divorced	7	5.0
Widowed	28	20.0
**Total**	**140**	**100.0**
**Education**		
No formal education	9	6.4
Primary	36	25.8
Post-primary	95	67.8
**Total**	**140**	**100.0**
**Occupation**		
Trading	43	30.7
Public servant	31	22.1
Farming	22	15.7
Artisan	11	7.9
Driver	6	4.3
Clergy	4	2.9
Retired	23	16.4
**Total**	**140**	**100.0**

*n*, Given as number of patients.

Ninety-eight (70.0%) patients had been hypertensive for less than 3 years, whilst 42 (30.0%) had been hypertensive for 3 years or more. Of the 49 patients that had good BP control, 29 (59.2%) had been hypertensive for 3 years or more whilst 20 (40.8%) had been hypertensive for less than 3 years. The difference between the two groups was statistically significant (*χ*^2^ = 3.18; df = 1; *p* - value = 0.042) (Table 2).

**TABLE 2:** Relationship between duration of antihypertensive medication and blood pressure control.

Duration of medication	Controlled		Not controlled	
	
	*n*	%	*n*	%
< 3 years	20	40.8	78	85.8
≥ 3 years	29	59.2	13	14.2

**Total**	**49**	**100.0**	**91**	**100.0**

*n*, Given as number patients. *χ*^2^ = 3.18; df = 1; *p*-value = 0.042

The mean BP at the recruitment visit (170.6 ± 10.4 per100.8 mmHg ± 12.3 mmHg) was higher than the mean BP at the end of study visit (130.6 ± 4.4 per 80.3 mmHg ± 8.6 mmHg). This difference was statistically significant (*t* = 4.26; *p*-value = 0.036).

Sixty (42.9%) out of 140 patients were adherent, whilst 80 (57.1%) were not adherent. Of the 49 patients who had good BP control, 45 (91.8%) were adherent whilst 4 (8.2%) were not adherent. BP control was significantly higher in those that adhered to antihypertensive medication compared with non-adhering patients (*χ*^2^ = 5.12; df = 1; *p*-value = 0.03) (Table 3).

**TABLE 3:** Association between adherence to medication and blood pressure control.

Medication pattern	Controlled		Not controlled	
	
	*n*	%	*n*	%
Adherence	45	91.8	15	16.5
Non-adherence	4	8.2	76	83.5

**Total**	**49**	**100.0**	**91**	**100.0**

*n*, Given as number patients. *χ*^2^ = 5.12; df = 1; *p*-value = 0.03

Blood pressure control by number of antihypertensive medications (Table 4). The difference between taking one antihypertensive medication and taking more than one antihypertensive medication was statistically significant (*χ*^2^ = 5.11; df = 2; *p*-value = 0.04) (Table 4).

**TABLE 4:** Blood pressure control by number of antihypertensive medications

Number of drugs	Controlled		Not controlled	
	
	*n*	%	*n*	%
One drug	9	18.4	52	57.1
Two drugs	17	34.7	24	26.4
T ≥Three drugs	23	46.9	15	16.5

**Total**	**49**	**100.0**	**91**	**100.0**

*n*, Given as number patients. *χ*^2^ = 5.11; df = 2; *p*-value = 0.04.

The most common reason proffered for non-adherence to medications by the hypertensive patients was forgetfulness (32.4%). Other reasons included feeling of well-being (25.6%), lack of funds (25.6%), advice by spiritual leaders (8.7%) and herbal remedies (7.7%). This marginal difference was statistically significant (*χ*^2^ = 6.03; df = 4; *p*-value = 0.046) (Table 5).

**TABLE 5:** Reasons for non-adherence to medication (*N* = 80).

Reason for non-adherence to medication	Yes		No	
	
	*n*	%	*n*	%
Forgetfulness	71	32.4	9	5.0
Feeling of well-being and cure	56	25.6	24	13.3
Lack of funds (to purchase drugs)	56	25.6	24	13.3
Advice by spiritual leaders	19	8.7	61	33.6
Herbal remedies	17	7.7	63	34.8
Total responses	219	100.0	181	100.0

Note: Multiple responses were recorded for some patients; percentages represent proportion of responses obtained. *n*, Given as number patients. *χ*^2^ = 6.03; df = 4, *p*-value = 0.046.

## Discussion

The BP control rate in this study of 35.0% is higher than the 24.2% reported in Port Harcourt, Rivers State, south-south Nigeria,^10^ 31.4% reported in Osogbo, south-west Nigeria,^11^ and 33% reported in Kano, Northern Nigeria.^14^ However, the BP control rate in this study is lower than the 35.8% reported in Ilorin, North Central Nigeria^13^ and 40.0% reported in South Africa.^25^ The findings of this study corroborate previous reports that adequate BP control rates are low in Nigeria and occur only in a fraction of treated hypertensives.^9, 10, 11, 12, 23, 14^ These disparities are probably due to a milieu of limited healthcare facilities, misconceptions and health beliefs about hypertensive disorder.^26^

Although adequate BP control does not completely obviate the risk of developing complications of hypertension, experience from developed countries has left no doubt that there is no magic bullet for adequate BP control amongst hypertensives.^2, 3^ Although low BP control rates are due to multiple factors, in this study this may not be unconnected from patient factors of adherence (forgetfulness) to medication amongst others. Clinicians attending to adult Nigerians with primary hypertension should therefore ask specific questions in order to uncover medication problems. in the early stages, since hypertension is a silent killer that usually shows no specific symptoms. This will enable adult Nigerians with primary hypertension to benefit from the life-saving benefits of antihypertensive medications. This study has shown that BP control was significantly higher amongst patients that adhered to their medication. The adherence rate of 42.9% in this study is lower than that of 45.0% reported in Kano, Northern Nigeria^14^ and of 43.0% reported in Lake City in the USA.^27^ The findings of this study have demonstrated the contribution of adherence in BP control amongst the study population. Although there may be other inter-patient variability amongst the study population that may contribute to poor BP control despite adherence to medications, efforts should therefore be made by clinicians to ensure that their adult hypertensive patients adhere to their antihypertensive medications. Clinicians inquiring about medication adherence during their encounters with adult hypertensive patients remains imperative in the drive to lower BP to goal levels. Without regular feedback on medication adherence between clinicians and hypertensive patients, a patient with poor BP control may never achieve their BP target.

Blood pressure control was significantly associated with duration of hypertension. The finding of a higher BP control rate amongst patients who had had their hypertensive condition for three or more years could be attributed to their increasing contact with the hospital and other health facilities where the importance of consistent antihypertensive medication is emphasised. These patients with longer duration of hypertensive medication, by virtue of their frequent contact with health facilities, are also more likely to be aware of the fatal and non-fatal complications of uncontrolled BP. In addition, it could be a reflection of wider social interaction with other hypertensive patients who are adherent to antihypertensive medication. This result supports the observation by Nichol Venturin and Sung^28^ on critical evaluation of literature on medication compliance that adherence to medication tends to increase over time for patients on chronic medication such as hypertensive patients.

This study has also shown that BP control is related to the number of antihypertensive medications. This finding is similar to reports from other parts of Nigeria,^14, 29^ and is consistent with a rational approach for treatment of hypertension in adult Black hypertensives.^2^ This could be a reflection of the pathophysiological mechanisms involved in Black hypertensive patients,^2^ and provides additional evidence on the growing need for combination therapy amongst the study population. This therefore calls for wider application of evidence-based hypertensive management guidelines in addition to diverse actions against hypertension, because adequate control of BP is associated with more health benefits and less risk of adverse cardiovascular events.^4, 15^

Although low BP control rates are polyfactorials, this study observed that the most common reason proffered for by non-adhering hypertensive patients was forgetfulness. This could be attributed to life-competing psychosocial demands amongst the study population, who were mainly traders. This finding has buttressed the report that psychosocial variables are additional barriers to adherence to antihypertensive medication.^30^ Non-adherence resulting from forgetfulness, amongst other reasons, could lead to poor BP control and higher costs in physicians’ visits. This has also shown that forgetting to take antihypertensive medication is not a problem that adult patients outgrow. Awareness of this by clinicians is crucial for optimal care of adult Nigerian hypertensive patients in the study area. Clinicians should therefore be aware of these subtleties, because they can influence the quality and quantity of care delivered to these patients.

### Implications of the study

Despite the publication of major clinical trials on the benefits of attaining target BP amongst hypertensives, the actual BP control rates in adult hypertensive Nigerians remains a management challenge. Suffice it to say that substantial risk of cardiovascular events still exists, despite treatment of hypertension, depending on the difference between achieved BP and target BP. However, the bulk of mortality associated with cardiovascular disease is principally attributed to unsatisfactory BP control resulting in target organ damage. Hence there is a need to determine the magnitude of BP control amongst adult hypertensives in the study centre as part of the search for an effective intervention to prevent complications of hypertension amongst the study population. This study therefore provides additional evidence on the growing problem of BP control rates in Nigeria, and calls for improved management of hypertension through adherence counselling as well as strengthening the quality of care. This will enable adult hypertensive Nigerians to benefit from the life-saving advantages of goal BP control.

## Limitations of the study

The limitations imposed by the self-reported and qualitative measure of adherence for the study are recognised by the authors. Also, the sample size for the study was relatively small, but was more than the minimum estimated sample size for the study and was the number of patients seen within the proposed duration of the study.

The questionnaire was administered by three resident postgraduate medical doctors. This could have introduced interviewers’ influence and bias, since some patients couldn't clinically give true responses to questions related to adherence in the presence of their physicians. However, pre-testing of the questionnaire internally and externally using the same resident medical doctors did not reveal this limitation. In addition, the respondents were assured of confidentiality prior to the interview.

## Conclusion

The BP control rate amongst the study population was low. This may not be unconnected with low adherence amidst other factors determining target BP control. The most common reason for non-adherence was forgetfulness. This study urges consideration of adherence factors alongside the milieu of other factors driving goal BP control. The use of reminders such as an alarm system or beepers amongst other interventions to overcome forgetfulness is advocated.
